# Retraction: Initial insights into post-contrast enhancement in ultra-low-field MRI: Case Report

**DOI:** 10.3389/fnimg.2026.1816667

**Published:** 2026-03-11

**Authors:** Frontiers Editorial Office

Following publication, concerns were raised regarding data misrepresentation. Significant concerns were identified regarding the accuracy of the imaging data, including mislabeled figures. The authors were unresponsive to requests for the raw data for this study. Given extant concerns over the validity of the study, the article has been retracted.

This retraction was approved by the Chief Editors of Frontiers in Neuroimaging and the Chief Executive Editor of Frontiers. The authors have not responded to correspondence regarding this retraction.

Frontiers would like to thank Cesare Gagliardo for contacting the journal regarding the published article.

